# IRW (Ile–Arg–Trp) Alleviates DSS–Induced Intestinal Injury by Remodeling Gut Microbiota and Regulating Fecal SCFA Levels

**DOI:** 10.3390/nu15040953

**Published:** 2023-02-14

**Authors:** Yanquan Fei, Siying Li, Zaoyi Wang, Yong Ma, Jun Fang, Gang Liu

**Affiliations:** Hunan Provincial Engineering Research Center of Applied Microbial Resources Development for Livestock and Poultry, College of Bioscience and Biotechnology, Hunan Agricultural University, Changsha 410128, China

**Keywords:** IRW, oligopeptide, gut microbiota, *Odoribacter*

## Abstract

Inflammatory bowel disease (IBD) is a chronic disease of unknown etiology with a progressive and destructive course and an increasing incidence worldwide. Dietary peptides have a variety of biological functions and are effective anti-inflammatories and antioxidants, making them a prospective class of material for treating intestinal inflammation. Our study investigated the association between Ile–Arg–Trp (IRW), a dietary oligopeptide, and intestinal microbial changes during the relief of colitis using different concentrations of IRW. We found that IRW can significantly alleviate mouse colonic barrier damage caused by dextran sulphate sodium salt (DSS) and promote intestinal health. The results of microbial community composition showed that the relative abundance of Bacillota and *Lactobacillus* in the gut microbiota at different concentrations of IRW was significantly increased and that the abundance of *Bacteroides* was suppressed. Surprisingly, the relative abundance of *Odoribacter* also received regulation by IRW concentration and had a positive correlation with acetic acid. IRW at 0.02 mg/mL and 0.04 mg/mL significantly altered the abundance of Bacillota, *Odoribacter*, and *Lactobacillus*.

## 1. Introduction

IBD is a systemic, multifactorial, complex, chronic disease with recurrent and remission periods [1,2,3]. Patients with IBD are at high risk of increased intestinal permeability, which can induce translocation of lipopolysaccharide, a product of intestinal bacteria, into the blood circulation, triggering an acquired immune response and exacerbating systemic inflammation [4]. Currently, the most common strategies for treating IBD include immunosuppressants, corticosteroids, and anti-tumor necrosis factor (*TNF*) drug therapy [5]. However, such approaches have significant side effects and are costly. Foodborne bioactive substances are being given increasing attention for the early treatment of IBD [6]. Recent studies have shown that the dietary polyphenol resveratrol [7], dietary fiber pectin polysaccharides [8], dietary brassica anthocyanins [9], and dietary protein ovalbumin [10] maintain intestinal barrier integrity, modulate microbiota homeostasis, and have anti-inflammatory properties.

Peptides of food protein origin have a wide range of biological functions and are used to various degrees in biomaterial development, the food industry, and peptide-based therapeutics [11,12]. The angiotensin-converting enzyme inhibitor Ile–Arg–Trp (IRW) is a food-derived oligopeptide that was identified in the egg protein ovotransferrin [13]. It inhibits angiotensin-II-induced oxidative stress, inflammation, and migration in vascular smooth muscle cells. It exhibits anti-hypertensive effects by regulating endothelial function, inhibiting vascular inflammation, and enhancing NO levels in endothelial cells [14]. As an antioxidant, 50μM IRW increased Caco-2 cell viability, reduced reactive oxygen species (ROS) production, and normalized mitochondrial membrane potential [15]. In human umbilical vein endothelial cells, IRW attenuated the *TNF*-*α*-induced inflammatory response and oxidative stress [16]. In addition, in terms of anti-inflammatory effects, IRW can increase adenosine triphosphate (ATP) gene expression and reduce the gene expression of inter-cellular adhesion molecule 1 (*ICAM-1*) and vascular cell adhesion protein 1 (*VCAM-1*) to reduce leukocyte recruitment in the vascular system, lower the risk of vascular inflammation, and destroy atherosclerosis [17].

In our previous studies [18,19], treatment of acute colitis in mice with IRW at a concentration of 0.03% promoted a normalization of the ratio of Bacillota (Firmicutes) and Pseudomonadota (Proteobacteria) and an increase in the levels of *Lactobacillus*, *Anaerotruncus*, *Oscillibacter*, and *Ruminiclostridium*. Nevertheless, whether there is a concentration dependence between IRW and microorganisms in colitis has not been thoroughly investigated. Therefore, this study dosed DSS-fed mice with different concentrations of IRW to explore changes in gut microbe composition in colitis and to screen for differential microbes.

## 2. Materials and Methods

### 2.1. Animal

Female C57BL/6J mice (6 weeks old, 18 ± 2 g) were purchased from Hunan Slaughter Jingda Laboratory Animal Co. Ltd. (Changsha, China). All experimental animals and experiments were approved and met the criteria of the ethics committee of Hunan Agricultural University. Mice were housed in a pathogen-free room with a temperature of 23 ± 2 °C, humidity of 50 ± 5%, and a light/dark cycle of 12 h. Mice were randomly divided into 7 groups (*n* = 5; no significant difference in body weight): a control group, a DSS group, a 0.02 IRW group (0.02 mg/mL IRW, Ontores, Hangzhou China), a 0.04 IRW group, a 0.06 IRW group, a 0.08 IRW group, and a 0.1 IRW group. After one week of acclimatization, the control and DSS groups were fed normal drinking water. The other five groups were fed the corresponding IRW concentrations in their drinking water. After seven days, the drinking water was replaced with 2.5% DSS, which was given for seven days in the remaining six groups (except the control group). Sampling was performed after three days. Damage to the colon, intestinal microorganisms, and fecal content of short-chain fatty acids (SCFAs) were measured.

### 2.2. Histopathological Analysis

Mouse colon tissue was dehydrated in ethanol, hyalurated in xylene, and embedded in paraffin blocks. The colonic paraffin blocks were cut into 5 µm thick sections. After baking and decolorization, the sections were stained with a periodic acid Schiff (PAS) staining kit (Wellbio, Shanghai, China) and hematoxylin–eosin (Wellbio, Shanghai, China). Microscopy (Motic, Xiamen, China) was used for observation after blocking.

### 2.3. Determination of SCFAs in Feces

The collected mouse feces were added to distilled water, shaken, mixed overnight at 4 °C, and centrifuged at 15,000 rpm for 20 min. The supernatant was mixed with 25% metaphosphoric acid at a volume ratio of 9:1 and left to react at room temperature for 4 h. The supernatant was filtered and added to a gas phase injection vial for gas chromatography–mass spectrometry (GC-MS, Agilent, USA) analysis. The analysis was performed using a free fatty acid phase (FFAP) column with high purity nitrogen as the carrier gas at a flow rate of 0.8 mL/min. The inlet temperature was 250 °C and the injection volume was 1 µL. The initial temperature was 60 °C, which was increased to 220 °C at 20 °C/min and maintained for 1 min. Acetic acid, propionic acid, isobutyric acid, butyric acid, isovaleric acid, and valeric acid standards (Sigma-Aldrich) were mixed at different concentrations and measured under the same conditions.

### 2.4. 16S rDNA Pyrophosphate Sequencing

The contents of all mouse colon segments are taken at the end of the experiment and ground beads and lysate are added, shaken and mixed and centrifuged to remove the supernatant. Total DNA was extracted from the bacteria using the NucleoSpin 96 Soi (MN, Germany) DNA extraction kit. Full-length 16S rRNA was amplified using primers 338F (5′-ACTCCTACGGGAGGCAGCA-3′) and 806R (5′-GGACTACHVGGGTWTCTAAT-3′). DNA was purified using an OMEGA DNA Purification Kit (Omega, USA) and recovered by gel cutting using a Monarch DNA Gel Recovery Kit (NEB, UK). The DNA was sequenced using a two-step library-building method. The raw data were then spliced (FLASH version 1.2.11, CBCB, Maryland, USA), the spliced sequences were quality filtered (Trimmomatic, version 0.33, USADELLAB.org, Jülich, Germany), and chimeras were removed (UCHIME, version 8.1, Robert C Edgar, CA, USA) to obtain high-quality tag sequences. The species were annotated and taxonomically analyzed using the Silva database.

### 2.5. Statistical Analysis

GraphPad Prism 9 (GraphPad Software Inc., San Diego, CA, USA) software and SPSS 22 software (SPSS Inc., Chicago, IL, USA) were used to analyze the data. The statistical differences between the groups were determined using one-way analysis of variance (ANOVA) and Tukey’s multiple comparison tests.

## 3. Results

### 3.1. Effect of Different Concentration Gradients of IRW on Body Weight and Immune Organ Index in Mice with Colitis

**Figure 1:**
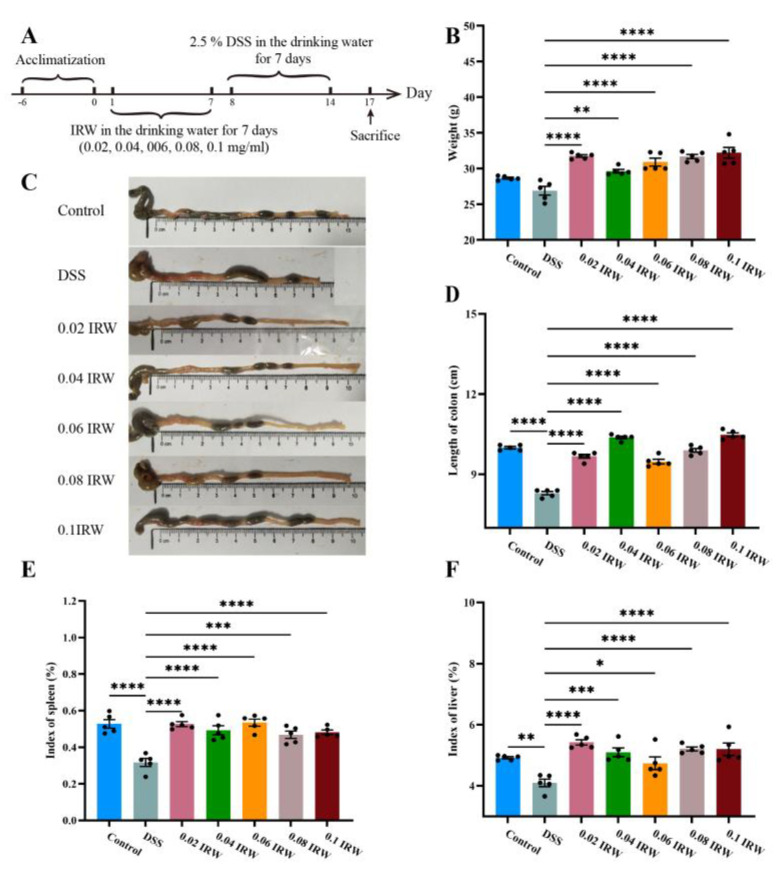
Effects of different concentrations of IRW on weight, colon length and visceral weight in mice: (**A**) experimental process, (**B**) mouse weights, (**C**) photos showing colon length, (**D**) colon length statistics, (**E**) index of spleen, and (**F**) index of liver (*n* = 5); * indicates *p* < 0.05; ** indicates *p* < 0.01; *** indicates *p* < 0.001; **** indicates *p* < 0.0001.

As shown in Figure 1A,B, the body weight of DSS mice increased after receiving the IRW treatment, and there was a significant difference compared with the DSS group (*p* < 0.01). Among them, the 0.02 and 0.1 IRW groups had the most weight recovery (*p* < 0.0001). The colonic contents of the DSS-treated mice exhibited fluffy pieces with ragged edges and a mushy stool, while the IRW-treated mice had soft blobs with clear-cut edges (Figure 1C). Meanwhile, the shortening of colonic length caused by the toxic effects of DSS was significantly alleviated by the IRW intervention (*p* < 0.0001). IRW at a concentration of 0.1 mg/mL had the best effect on the length recovery of the colon (Figure 1D). This indicates that IRW has a somewhat preventive effect in the context of colitis. The immune organ index provides good visualization of the function of the immune organs. As illustrated in Figure 1E,F, the liver and spleen indices of the IRW-dosed mice were significantly higher than those of the DSS group (*p* < 0.05) and similar to those of the control group. These findings suggest that IRW alleviates immune organ atrophy caused by DSS to a certain extent.

### 3.2. Protective Effect of Different Concentration Gradients of IRW on Intestinal Barrier in Mice with Colitis

**Figure 2:**
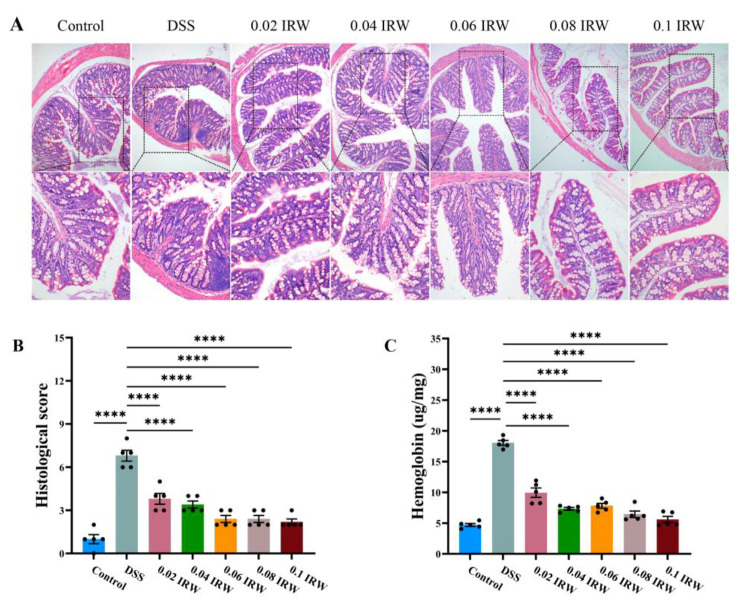
Effects of different concentrations of IRW on colonic morphology in mice: (**A**) HE staining of the colon, (**B**) histological score of the colon, and (**C**) colon tissue hemoglobin content (*n* = 5); **** indicates *p* < 0.0001.

Figure 2A shows the abnormal inflammatory infiltrates (basal lymphocyte aggregates, crypt abscess) caused by DSS, along with its crypt atrophy and widened opening, with surface irregularity of the mucosa and villi-like changes. These were effectively prevented after receiving different concentrations of IRW interventions. No inflammatory infiltration occurred in the colons of any mice receiving an IRW intervention. The histological scores were significantly lower in the IRW groups than in the DSS group (*p* < 0.0001; Figure 2B). Among them, 0.08 and 0.1 mg/mL IRW maintained the crypt structure and kept the mucosal surface flat (Figure 2A). Compared with the DSS group, the hemoglobin content in the feces of mice in the IRW groups was significantly lower, effectively preventing the symptoms of internal hemorrhage of the colon in acute colitis caused by DSS (*p* < 0.0001; Figure 2C).

### 3.3. Inhibitory Effects of Different Concentration Gradients of IRW on Intestinal Mucosal Destruction and Goblet Cell Exhaustion in Mice with Colitis

**Figure 3:**
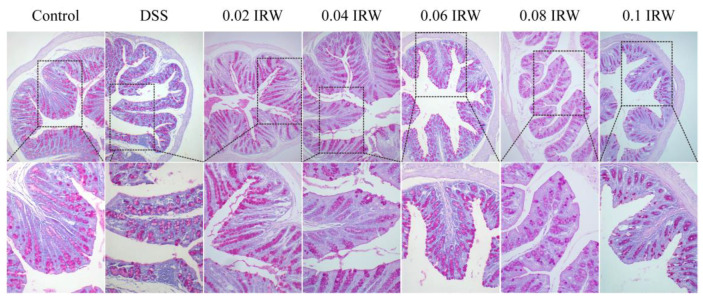
Pathological section of PAS staining (black box represents magnified area).

The mucin secreted by goblet cells is part of the intestinal barrier and forms a physical barrier with digestive juices and antimicrobial substances secreted by normal epithelial cells. The mucus layer effectively prevents direct contact of bacteria and lipopolysaccharide (LPS) with intestinal epithelial cells. As shown in Figure 3, PAS staining was used to evaluate the integrity of the colonic mucus layer. The colonic mucus layer was intact and continuous in the control group. By contrast, in the DSS group, the structure of goblet cells was disrupted, the mucus layer was fragmented, and the mucus distribution was drastically reduced. The disruption of the intestinal barrier further aggravated intestinal damage and increased inflammation. However, this situation was greatly improved in the colons of DSS mice that received different concentrations of the IRW intervention. IRW significantly inhibited the depletion of goblet cells and maintained the integrity of the mucus layer as well as the uniform distribution of upheld mucus.

### 3.4. Different Concentration Gradients of IRW Regulate Gut Microbiota in Mice with Colitis

**Figure 4:**
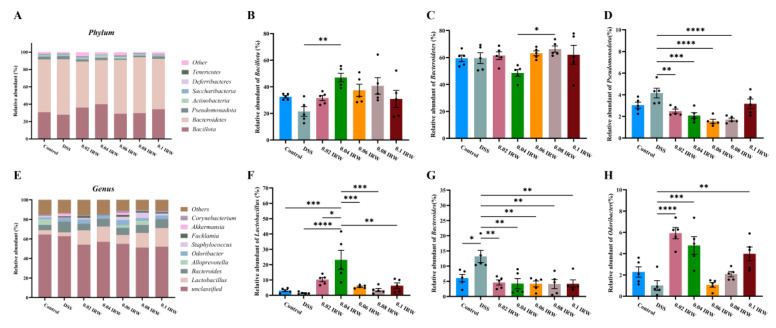
Microbial composition of the mouse colon: (**A**) microbial composition at the phylum level, (**B**) relative abundance of Bacillota, (**C**) relative abundance of Bacteroidota, (**D**) relative abundance of Pseudomonadota, (**E**) microbial composition at the genus level, (**F**) relative abundance of *Lactobacillus*, (**G**) relative abundance of *Bacteroides*, (**H**) relative abundance of *Odoribacter* (*n* = 5); * indicates *p* < 0.05; ** indicates *p* < 0.01; *** indicates *p* < 0.001; **** indicates *p* < 0.0001.

To better understand the effects of different concentrations of IRW on the composition of the gut microbiota of DSS-fed mice, the most abundant microbes in each group were studied at the phylum and genus levels. At the phylum level, Bacillota, Bacteroidota, Pseudomonadota, and Actinobacteriota were the most dominant phyla in the mouse intestinal microbiota. The highest total abundance of the four dominant phyla was 97.56% in the 0.08 IRW group, followed by 96.30% in the 0.04 IRW group and 96.29% in the 0.1 IRW group (Figure 4A). The relative abundance of Bacillota in the intestines of mice receiving IRW interventions was significantly higher than in the DSS group, with the highest value being 46.97% in the 0.04 IRW group (*p* < 0.01; Figure 4B) and the lowest relative abundance being of Bacteroidota in the 0.04 IRW group (Figure 4C). The relative abundance of Pseudomonadota in the intestines of mice receiving IRW interventions was significantly lower than in the DSS group (*p* < 0.05), with the lowest relative abundance being in the 0.06 IRW group (Figure 4D). The dominant genera were mainly *Lactobacillus*, *Bacteroides*, *Odoribacter,* and *Alloprevotella*, with relative abundances of 11.70%, 7.82%, 3.57%, and 1.85%, respectively (Figure 4E). Among the intestinal microorganisms of DSS mice receiving IRW interventions, the highest relative abundance of *Lactobacillus* compared to the DSS group was 23.11% in the 0.04 IRW group (*p* < 0.0001; Figure 4F). By contrast, the relative abundance of *Bacteroides* in all the IRW groups was significantly lower than in the DSS group (*p* < 0.01; Figure 4G). Surprisingly, the relative abundance of *Odoribacter* in the 0.02 IRW (*p* < 0.0001) and 0.04 IRW (*p* < 0.001) groups showed a significant increase compared to the DSS group (Figure 4H).

### 3.5. Effect of Different Concentration Gradients of IRW on the Concentration of SCFAs in the Colon of Mice with Colitis

**Figure 5:**
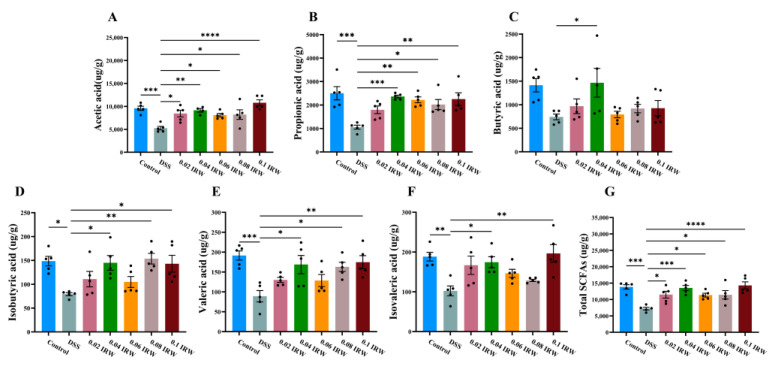
The fecal SCFA content. The content of (**A**) acetic acid; (**B**) propionic acid; (**C**) butyric acid; (**D**) isobutyric acid; (**E**) valeric acid; (**F**) isovaleric acid; (**G**) total SCFAs; * indicates *p* < 0.05; ** indicates *p* < 0.01; *** indicates *p* < 0.001; **** indicates *p* < 0.0001.

SCFAs lower the pH of the colon and thus inhibit pathogen colonization and regulate the function of the intestinal mucosal barrier. Therefore, to determine the effect of IRW on the intestinal microbiota, the contents of various types of SCFAs and total SCFAs in mouse feces were measured. DSS severely downregulated the content of various SCFAs in mouse feces, with significant decreases in acetic acid, propionic acid, valeric acid, and total SCFAs compared to the control group (*p* < 0.001; Figure 5A,B,E,G). All types of SCFA content increased appreciably after receiving the IRW intervention. In particular, both acetic acid and total SCFA content were significantly higher in the IRW groups (albeit at different concentrations) compared to the DSS group (*p* < 0.05; Figure 5A,G). Surprisingly, acetic acid (*p* < 0.01), propionic acid (*p* < 0.001), butyric acid (*p* < 0.05), isobutyric acid (*p* < 0.05), valeric acid (*p* < 0.05), isovaleric acid (*p* < 0.05), and total SCFA (*p* < 0.001) levels were significantly upregulated at the 0.04 IRW concentration compared to the DSS group.

### 3.6. Analysis of the Association between SCFAs and Microorganisms at the Genus Level

**Figure 6:**
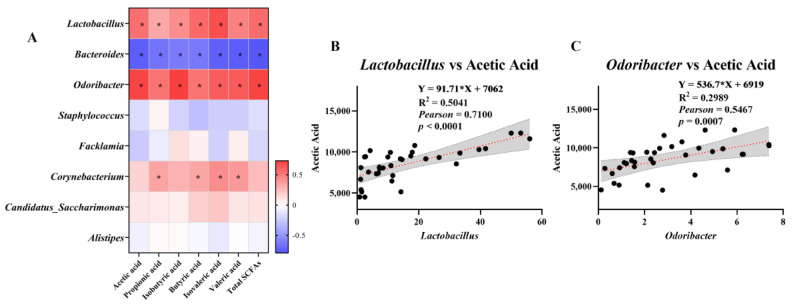
Correlation analysis: (**A**) heatmap of the correlation between fecal SCFAs and microbes, (**B**) positive correlation between *Lactobacillus* and acetic acid, and (**C**) positive correlation between *Odoribacter* and acetic acid; * indicates *p* < 0.05.

To investigate whether IRW alleviates the intestinal inflammatory response by modulating specific microorganisms and SCFA levels, Pearson correlations between key species in the fecal microbiota and SCFAs were analyzed (Figure 6A). *Lactobacillus* and *Odoribacter* were significantly positively correlated (*p* < 0.05) with acetic acid, propionic acid, isobutyric acid, butyric acid, isovaleric acid, valeric acid, and total SCFA content, while *Bacteroides* was significantly negatively correlated (*p* < 0.05). *Corynebacterium* was positively correlated with propionic acid, butyric acid, valeric acid, and isovaleric acid, respectively (*p* < 0.05). Pearman correlation showed a highly significant positive correlation between acetic acid and *Lactobacillus* and *Odoribacter* (*p* < 0.001; Figure 6B,C), which indicates that, in this study, IRW increased the abundance of *Lactobacillus* and *Odoribacter* in the colon while increasing the level of acetic acid in feces.

## 4. Discussion

We conducted this study to determine the impact of IRW at various doses on DSS–Induced colonic inflammation. Overall, the colons of mice treated with different concentrations of IRW showed a complete histological structure compared with the DSS–Induced colons. Based on the PAS results, IRW can help goblet cells in the colonic epithelial tissue of DSS mice secrete mucus and participate in the mucosal barrier repair mechanism. Previous findings indicate that IRW upregulates angiotensin-converting enzyme 2 (*ACE2*) levels in the mesentery of spontaneously hypertensive rats, which helps reduce vascular inflammation and restore vascular epithelial tissue permeability [20]. In this study, the reduced hemoglobin content in the feces of mice after IRW intervention also reflected this process. Bioactive peptides are also promoters of intestinal mucus-producing cells that induce mucin expression. They increase the number of small intestinal goblet cells and Paneth cells to protect the intestinal lumen from destructive substances [21].

Disturbances in the composition of gut microbes usually accompany host intestinal inflammation, with an increase in Pseudomonadota and *Bacteroidetes*. In our experiment, IRW supplementation significantly reduced the abundance of Pseudomonadota and *Bacteroides*. *Bacteroides* are usually symbiotic with the host as potential probiotics but can become opportunistic pathogens under certain conditions [22]. For example, the abundance of *Bacteroides* in fecal samples from celiac disease patients with an impaired intestinal barrier is abnormally high [23,24]. This may be due to some anaerobic pathogens in *Bacteroides*, including *Bacteroides fragilis*. These changes in *Bacteroides fragilis* abundance may have potential biomarkers for predicting colorectal inflammation-related diseases [25]. We also note that the 0.04 IRW group had the highest relative abundance values of Bacillota and *Lactobacillus*. *Lactobacillus* restores intestinal barrier function by regulating tight junction proteins through the nuclear factor kappa-B (*NF-κB*) signaling pathway [26,27]. This structural change in the gut microbiota is consistent with a symptom of inflammatory remission. Surprisingly, we also found that the 0.04% IRW treatment significantly increased *Odoribacter. Odoribacter*, a member of the order Bacteroidales, is a widespread component of the human intestinal microbiota that produces SCFAs [28]. Decreased *Odoribacter* abundance has been associated with a variety of microbiota-related illnesses, including non-alcoholic fatty liver disease, cystic fibrosis, and IBD [29]. Moreover, *Odoribacter* was identified in the intestine of a mouse strain Tak1 ^ΔM/ΔM^, which is effective against intestinal inflammatory colon cancer and was also verified to induce the differentiation of immunosuppressive Th17 cells with a highly significant protective effect against colitis and colon cancer [30]. In a clinical trial of fecal transplantation in ulcerative colitis, it was discovered that *Odoribacter* is a type of metastatic bacteria that shapes mucosal immunity by increasing *IL-10* expression and the production of SCFAs, resulting in an increase in regulatory T cells. The results of treatment of intestinal inflammation, such as fecal bacteria transplantation, showed a significant increase in *Odoribacter* abundance, which is consistent with our experimental results [28]. In this study, the relative abundance of *Odoribacter* in mice after receiving the IRW intervention was not reduced by the effect of DSS. In particular, the relative abundance of *Odoribacter* increased relative to the control group at IRW concentrations of 0.02 mg/mL and 0.04 mg/mL.

SCFAs are important substrates for maintaining the activity of intestinal epithelial cells, and their levels in the colon are closely related to intestinal barrier function [31]. Studies have found that SCFAs, including acetate and propionate, promote the renewal of intestinal epithelial cells [32] and inhibit the production of tumor necrosis factor-α and interferon-γ [33]. Our results showed that IRW significantly increased the content of SCFAs in feces and alleviated DSS–Induced colitis in mice. The results of microbial community composition and SCFAs indicate that 0.04% IRW had the best therapeutic effect. This is mostly consistent with the results of our previous study, which showed that 0.03% IRW can reduce the inflammatory infiltration of immune cells, promote the recovery of colon length, and slow down the process of intestinal inflammation [19]. This may be because IRW contains tryptophan [16]. As an essential aromatic amino acid, tryptophan can be metabolized by the intestinal microbiota via the indole pathway [34]. Its metabolites provide ligands to the host and promote IL-22 production, which regulates the release of antimicrobial peptides from epithelial cells and regulates intestinal microbiota to alleviate intestinal inflammation [35]. Therefore, 0.04% may be the optimal concentration. When the IRW concentration is too high, the amino acids obtained by hydrolysis cannot be used by the host. Excessive accumulation of isoleucine, arginine, and tryptophan from IRW catabolism in the host will increase the burden on the metabolic system, leading to metabolic disorders [36,37,38].

## 5. Conclusions

In conclusion, different concentrations of IRW ameliorated morphological damage to the colon caused by DSS. IRW treatment remodeled the intestinal microbial community composition and significantly increased fecal SCFA levels in mice. IRW at 0.02 mg/mL and 0.04 mg/mL significantly altered the abundance of *Odoribacter*, Bacillota, and *Lactobacillus*.

## Data Availability

All sequence data (16S) obtained from this study are available in the NCBI SRA database under the study numbers PRJNA901068.
